# Development of an mHealth Intervention for Reducing Sedentary Behavior in Older Adults: Delphi Study

**DOI:** 10.2196/83302

**Published:** 2026-06-11

**Authors:** Siqing Chen, Xiaofei Nie, Jiekai Yang, Yaqin Li, Lili Yang

**Affiliations:** 1 Nursing Department, Sir Run Run Shaw Hospital, Zhejiang University School of Medicine, 3 East Qingchun Road, Hangzhou, Zhejiang, 310016, China, 86 13429168765; 2 Department of Epidemiology, Harvard T.H Chan School of Public Health, Boston, MA, United States; 3 School of Nursing, Wenzhou Medical University, Wenzhou, China; 4 Department of Biobehavioral Sciences, Teachers College, Columbia University, New York, NY, United States

**Keywords:** older adults, sedentary behavior, mobile health, Delphi technique, China

## Abstract

**Background:**

Sedentary behavior among older adults is a major public health concern, contributing to the increased risk of chronic diseases and functional decline. With aging populations worldwide, prolonged sitting time (averaging up to 13 h/d in older adults) has been independently associated with cardiovascular disease, metabolic disorders, cognitive decline, and all-cause mortality. Mobile health (mHealth) interventions offer a promising approach to address this issue. However, there remains a lack of evidence-based, systematically developed mHealth programs specifically targeting sedentary behavior in older populations.

**Objective:**

This study aimed to develop an mHealth intervention program for reducing sedentary behavior in older adults using the Delphi consensus method.

**Methods:**

Guided by the Behavior Change Wheel framework, a preliminary mHealth intervention was developed using a combination of qualitative and quantitative methods, including a comprehensive literature review, clinical guidelines analysis, qualitative interviews, and a cross-sectional survey. The intervention was then refined through 2 rounds of Delphi surveys with 16 multidisciplinary experts in geriatric care, behavioral science, and health promotion. Consensus criteria were predefined as mean importance score >3.5 and coefficient of variation ≤0.25 on a 5-point Likert scale.

**Results:**

Both Delphi rounds achieved 100% response rates, with high expert authority coefficients (Cr=0.900 for Round 1 and Cr=0.907 for Round 2). The Kendall coordination coefficients (Kendall W) were 0.151 (*P*<.001) and 0.214 (*P*=.001) for the 2 rounds, respectively. Following 2 rounds of expert consultation, a total of 27 intervention items were finalized, comprising 3 core components addressing capability (eg, knowledge provision and behavioral skills training), opportunity (eg, social support and environmental restructuring), and motivation (eg, goal-setting, feedback, and incentives) factors influencing sedentary behavior.

**Conclusions:**

This study developed a theoretical framework-based, consensus-driven mHealth intervention program for reducing sedentary behavior in older adults. The intervention uniquely integrates the Behavior Change Wheel framework with expert validation, offering a comprehensive approach that simultaneously targets capability, opportunity, and motivation. The findings provide a structured foundation for future feasibility testing and effectiveness evaluation of mHealth interventions in aging populations. Future researchers should translate the developed mHealth intervention into an adaptive mHealth platform, followed by pilot testing and large-scale randomized controlled trials to evaluate its feasibility and effectiveness in real-world settings.

## Introduction

Sedentary behavior, defined as any waking activity characterized by an energy expenditure ≤1.5 metabolic equivalents while in a sitting, reclining, or lying posture [1], has emerged as a critical public health concern among older adults. With age-related physiological decline, increased prevalence of chronic diseases, and reduced social participation, older adults exhibit alarmingly prolonged sedentary time, averaging up to 13 hours per day [2]. This excessive sedentary behavior has been robustly associated with a spectrum of adverse health outcomes, including cardiovascular diseases, metabolic disorders such as obesity and type 2 diabetes, accelerated cognitive decline, and even increased all-cause mortality [3-5].

The rapid advancement of mobile health (mHealth) technologies presents a transformative opportunity for promoting behavioral change in this population [6,7]. mHealth interventions offer unique advantages, including real-time monitoring, personalized feedback, and scalable delivery, making them particularly suitable for addressing sedentary behavior in community-dwelling older adults [6,8]. However, current mHealth interventions in China remain fragmented, lacking theoretically grounded, systematically developed programs specifically targeting sedentary behavior reduction in aging populations. This critical gap underscores the urgent need for an evidence-based, theoretically informed intervention framework that integrates cutting-edge behavioral science with practical implementation considerations.

The Behavior Change Wheel (BCW) framework was applied in our previous systematic review, meta-analysis, and analysis of relevant clinical guidelines to identify the comprehensive determinants of sedentary behavior. These findings provided the foundation for developing a preliminary mHealth intervention protocol. Building on this work, our goal was to refine the protocol into a final intervention through 2 rounds of expert Delphi consultation. By providing a structured and theoretically grounded approach to intervention development, this study seeks to contribute a foundational framework for future implementation and evaluation of mHealth strategies targeting sedentary behavior in aging populations.

## Methods

### Overview

This study was reported in accordance with the Conducting and Reporting of Delphi Studies (CREDES) guidelines [9] (Checklist 1).

### Establishment of the Research Team

The research team comprised multidisciplinary members with expertise in nursing, health behavior, behavioral science, and exercise physiology. The team collaboratively developed the preliminary intervention components, reviewed and revised the Delphi questionnaires, and analyzed expert feedback through iterative discussions. Data collection and analysis were conducted by the doctoral researcher under the supervision of senior team members. Detailed professional profiles of all team members are presented in Multimedia Appendix 1.

### Development of the Expert Consultation Questionnaire

The development of the mHealth intervention preliminary version followed a structured process based on the 3 phases and 8 steps of the BCW framework. A comprehensive literature review and meta-analysis [6], qualitative research [8], and a cross-sectional study [10] previously conducted by the author were conducted to identify key determinants of sedentary behavior in older adults. These studies helped identify components for the mHealth intervention targeting sedentary behavior. Evidence-based guidelines, including the Canadian Physical Activity Guidelines for Older Adults and World Health Organization (WHO) 2020 guidelines [11,12], were also incorporated. The Capability, Opportunity, Motivation–Behavior (COM-B) model and Theoretical Domains Framework (TDF) within the BCW were used to map key intervention functions and behavior change techniques (BCTs) to the identified barriers. Following discussions within the research team, the first-round Delphi questionnaire was developed.

The expert consultation letter comprises 5 key sections. The first section is a cover letter introducing the research objectives, the methodology for completing the consultation form, and the deadline for submission. The second section provides an overview of the intervention program, including its background, purpose, and theoretical foundation. The third section focuses on evaluating the intervention functions and BCTs. Experts are asked to rate the appropriateness and importance of each selected intervention function and BCT using a 5-point Likert scale (1=very inappropriate or unimportant to 5=very appropriate or important). A dedicated column is provided for experts to suggest modifications or additions if necessary. The fourth section presents an evaluation table for the intervention program components, adapted from prior quantitative and qualitative research. Experts assess the reasonableness and significance of key elements, including intervention facilitators, objectives, content, duration, delivery format, and outcome evaluation, using the same 5-point scale. While the consultation covers the overall framework of the intervention, specific details, such as the definition of sedentary behavior, associated risks, intervention duration, and frequency, will be supplemented through a review of relevant domestic and international guidelines, with final approval determined by the research team. The final section is a self-assessment questionnaire for experts, collecting information on their professional background, judgment criteria, and familiarity with the content. The judgment criteria are categorized into theoretical analysis (scored 0.3, 0.2, or 0.1), practical experience (0.5, 0.4, or 0.3), reference to literature (0.1 for all levels), and intuitive selection (0.1 for all levels). Familiarity with the content is rated on a scale from 0 (unfamiliar) to 1.0 (very familiar), with intermediate options of 0.2, 0.5, and 0.8.

### Expert Selection Process

Experts were recruited through a combination of author identification from the literature and peer recommendations. The research team identified individuals with demonstrated expertise in geriatric care, behavioral science, and health promotion based on their publications in the field. Additionally, peer recommendations from professionals in these areas were sought to ensure a diverse and knowledgeable panel.

### Demographic Characteristics of Consulting Experts

The selection of experts for the Delphi consultation was determined based on the study’s scope and feasibility, with reference to established literature recommending a panel size of 15 to 50 participants [13,14]. In this study, a total of 16 multidisciplinary experts were recruited across 2 rounds of consultation, adhering to predefined inclusion criteria and the principles of informed consent and voluntary participation. Eligible experts were required to meet the following qualifications: (1) specialization in geriatric nursing, behavioral health promotion, exercise physiology, clinical medicine, nursing, or related fields; (2) substantial clinical or research experience, including involvement in relevant research projects; (3) possession of at least a bachelor’s degree; and (4) holding an intermediate or higher professional title. Among the 16 experts who completed both consultation rounds, 1 was male and 15 were female. All held a bachelor’s degree or higher, including 5 with master’s degrees and 11 with doctoral degrees. A total of 87.5% (14/16) of the participants held associate senior or higher professional titles. Detailed demographic characteristics are presented in Multimedia Appendix 1.

### Expert Consultation Process

The Delphi expert consultation was conducted through both email and face-to-face questionnaire distribution to eligible experts over 2 rounds, with the aim of finalizing the intervention protocol. To ensure independence and anonymity, experts completed the questionnaires independently without group discussion during the process, and all responses were deidentified prior to analysis. Each round was spaced 2‐4 weeks apart, and experts were given a 2-week response window [13]. In the first round, experts rated the items and provided feedback, which the research team analyzed. Items with low ratings, high variability, or unclear feedback were revised, merged, or added based on expert suggestions. The second-round questionnaire, along with a summary of first-round feedback, was redistributed for reevaluation. Items were considered to have reached consensus if they met the criteria: mean importance score >3.5 and coefficient of variation (CV)≤0.25. Items not meeting these criteria were refined based on expert comments. After the second round, consensus was achieved, and the final intervention was confirmed.

The expert authority coefficient (Cr) was calculated as the average of 2 self-assessed measures: judgment basis (Ca) and familiarity (Cs). This coefficient provided an indication of expert reliability, with values above 0.80 suggesting high authority in the relevant field. Furthermore, the Kendall coordination coefficient (Kendall W) was used to measure the level of agreement among experts. The interpretation of the W value is as follows: W≥0.60 indicates strong consensus, 0.30≤W<0.60 indicates moderate consensus, and W<0.30 indicates weak consensus.

### Ethical Considerations

Approval was obtained from the Ethics Committee of the Fourth Affiliated Hospital of Zhejiang University School of Medicine (approval number K2023150). Informed consent was obtained from all participants prior to participation. All data were anonymized prior to analysis to protect participant privacy and confidentiality. No financial compensation was provided to participants.

### Statistical Analysis

Data were analyzed using SPSS 27.0 (IBM Corp). Descriptive statistics (proportions, %) were used for qualitative demographic data. Expert engagement was assessed via response rate, while expert authority and consensus were evaluated using the Cr, CV, and Kendall concordance coefficient [13].

## Results

### Expert Engagement and Authority

Expert engagement reflected their commitment to the study. Across both rounds, all 32 distributed questionnaires (16 per round) were returned (100% response rate). In the first round, 12 of 16 experts provided suggestions, decreasing to 7 in the second round. The authority coefficient (Cr) was calculated as Cr=(Ca+Cs)/2, where Ca (judgment basis) and Cs (familiarity) were derived from expert self-assessments. The Cr values for both rounds were 0.900 and 0.907, respectively (Table 1).

**Table 1.** Expert authority coefficients.

Round	Judgment basis (Ca)	Familiarity (Cs)	Authority coefficient (Cr)
1	0.944	0.856	0.900
2	0.944	0.869	0.907

### Consensus and Agreement Among Experts

Consensus was evaluated using mean importance scores and CVs. Across both rounds, mean scores ranged from 4.20‐5.00 (Round 1) to 4.44–5.00 (Round 2), with CVs≤0.25 (Round 1: 0‐0.22; Round 2: 0‐0.20), indicating strong agreement. Kendall W revealed significant concordance (Round 1: W=0.151, *P*<.001 and Round 2: W=0.214, *P=.*001; Table 2).

**Table 2.** Concentration and coordination degree of expert opinions.

Item	Kendall W	Chi-square (*df*)	*P* value
Round 1			
Overall	0.151	74.527 (33)	<.001
Round 2			
Overall	0.214	61.651 (32)	.001

### Modification and Finalization of Questionnaire Content

#### Overview

**Figure 1.**
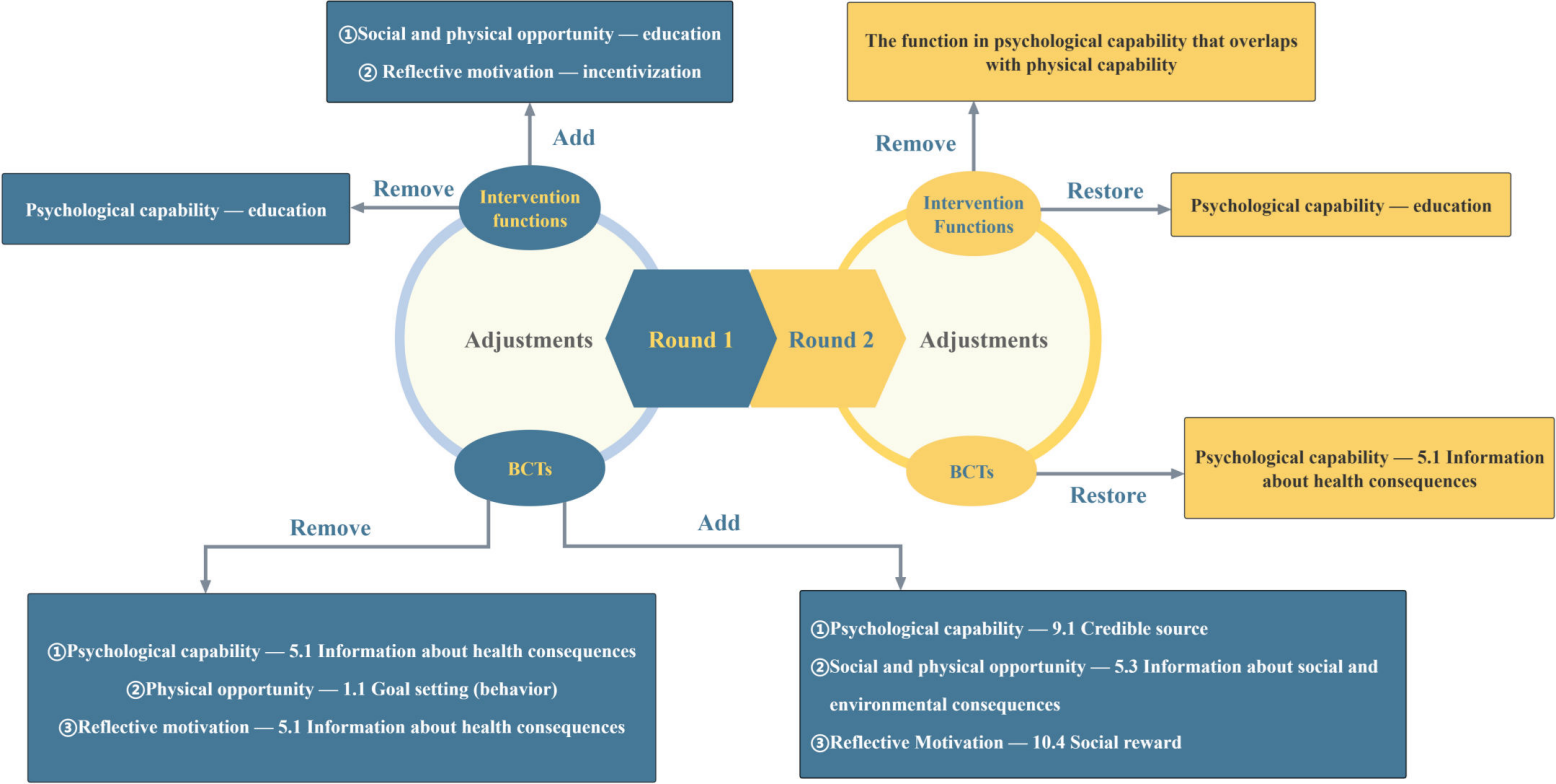
Adjustments to intervention functions and behavior change techniques in 2 Delphi rounds. BCT: behavior change technique.

Based on expert feedback from 2 rounds of Delphi consultation and discussions within the research team, several revisions were made to the intervention functions, BCTs, and the intervention protocol. In the first round, 1 intervention function was added, 3 BCTs were removed, and 1 additional BCT was deleted. In the second round, one more intervention function was added, another restored, and 3 BCTs were added while 2 were deleted. These modifications are summarized in Multimedia Appendix 2 and Figure 1, with a summary provided below:

#### Mapping Between Intervention Functions and BCTs

In the first round of expert consultation, experts pointed out that some BCTs were improperly matched with the 3 dimensions of the COM-B model. The research team accepted this suggestion, deleted the inappropriate BCT “1.1 Goal setting (behavior)” under “physical opportunity,” and added the “education” intervention function to eliminate barriers caused by limited physical opportunity from both theoretical and practical perspectives. Meanwhile, in response to experts’ proposals to add an “Incentive” function to the “Intention” dimension, the research team adopted the suggestion after discussion and supplemented corresponding BCTs; the BCT “5.1 Information about health consequences” deemed mismatched with the incentive function was deleted. In the second round of expert consultation, one expert further noted that skill training under psychological capability overlapped with physical capability. After discussion, the research team adopted this suggestion, deleted the relevant intervention function and its corresponding BCTs under “Psychological capability,” and restored the “education” intervention function and the BCT “5.1 Information about health consequences” that were deleted in the first round. This further enhanced the consistency and rationality of the intervention program in terms of the COM-B dimension classification.

#### Risk Perception and Educational Content

In the first round of expert consultation, experts recommended introducing authoritative sources to improve information credibility. The research team recognized that this suggestion would enhance the persuasiveness of risk perception, thus adding the BCT “9.1 Credible source” to strengthen intervention effects through forms such as videos from authoritative institutions. Additionally, addressing experts’ concerns about the single mapping of educational BCTs, the research team added the BCT “5.3 Information about social and environmental consequences” after discussion to supplement the explanation of social and environmental impacts of behaviors; however, the BCT “5.2 Salience of consequences” was not adopted as it did not fully meet the APEASE (Affordability, Practicability, Effectiveness and cost-effectiveness, Acceptability, Side-effects/safety, and Equity) criteria.

#### Social Support and Feedback Mechanisms

In the first round of expert consultation, some experts suggested introducing “3.2 Social support (practical).” After evaluating intervention costs, feasibility, and the APEASE criteria, the research team decided not to adopt this suggestion, as practical social support would incur high implementation costs and behavioral feedback lacked corresponding intervention pathways under the current barriers.

The figure illustrates the additions, removals, and restorations of intervention functions and BCTs based on expert feedback in Round 1 and Round 2.

Based on the opinions from 2 rounds of Delphi expert consultation and discussions within the research team, the overall modifications are as follows: after the first round of expert consultation, 1 item was deleted, 3 new items were added, and 17 items were revised; after the second round, 3 items were deleted, 1 item was added, and 5 items were revised. The experts’ detailed revision suggestions and consultation results are presented in Multimedia Appendix 2, summarized as follows.

#### Intervention Objectives

In the first round of expert consultation, experts suggested setting personalized and progressive reduction targets for screen time and clarifying the connotation of screen time. After discussion, the research team adopted this suggestion, specifying that screen time includes hours spent watching TV, using computers, and mobile phones, which would be mainly assessed through screen timers and questionnaires. Accordingly, the intervention objective was revised from “Reduce the total sedentary behavior duration (≤8 hours) and screen time (≤3 hours) of older adults, with reference to the Canadian 24-Hour Movement Guidelines” to “Reduce the total sedentary behavior duration (≤8 hours) of older adults and achieve personalized, progressive reduction in screen time (≤3 hours), with reference to the Canadian 24-Hour Movement Guidelines.” In the second round of expert consultation, experts pointed out that “personalized” and “progressive” were not specifically reflected in the objective statement. Given that further quantification of these elements in the intervention objective was impractical and they had already been concretely implemented in the intervention details, the research team decided to revert to the original expression in the Canadian 24-Hour Movement Guidelines. The final intervention objective was confirmed as “Reduce the total sedentary behavior duration (≤8 hours) and screen time (≤3 hours) of older adults, with reference to the Canadian 24-Hour Movement Guidelines.”

#### Intervention Implementers

Experts noted that fitness coaches in the implementation team had varying professional backgrounds and competence levels, and their professionalism in intervening in older adults’ sedentary behavior might be insufficient. They recommended that personnel with stronger professional backgrounds should lead the research implementation. The research team adopted this suggestion and revised “fitness coaches” in the original items to “experts from physical education colleges.” Furthermore, experts proposed that intervention implementers should not be limited to medical staff from geriatrics or rehabilitation departments and experts from physical education colleges, but should also include systematically trained researchers. Considering the indispensable role of researchers in the design, implementation, and evaluation of the program, the research team decided to include researchers in the scope of intervention implementers to enhance the scientificity and systematicness of intervention implementation.

#### Intervention Content

In response to experts’ comments that some items were vaguely expressed and lacked operability, the research team targeted revisions to the intervention content from 4 aspects: health information presentation, implementation and safety, forms of social support, and achievement incentive mechanisms.

1. Specification and contextualization of health information presentation: experts pointed out that some health information was presented in a single form and lacked contextual relevance. The research team made the following adjustments accordingly: in terms of presentation forms: Item 1.4 was revised from “providing only graphic guidelines for interrupting sedentary behavior” to include video demonstrations and voice guidance to improve learning outcomes; Item 1.5 was refined from the general “repeated practice of interrupting sedentary behavior” to “the platform triggers sedentary behavior reminders every 30 minutes to help individuals practice repeatedly until they proficiently master interruption skills”; To address the lack of contextualization in Items 1.7 and 3.7, the content was adjusted to present sedentary behavior risks and potential adverse health outcomes in different life scenarios (eg, entertainment, work, and aging) to enhance contextual relevance and targeting; As suggested by experts, Item 1.1 “Definition of sedentary behavior” was revised to “Overview of sedentary behavior” to ensure the systematicness and completeness of information; For Items 1.2 and 3.9 with unclear information sources, guidelines issued by authoritative institutions such as the WHO were explicitly cited to improve the scientificity and reliability of information sources.
2. Optimization of implementation methods and safety: experts believed that some intervention measures still had room for improvement in professionalism and safety. The research team adopted relevant suggestions and made revisions: Item 1.8 adjusted “fitness coaches” to “experts from physical education colleges” (with stronger professional backgrounds), and proposed personalized programs considering physical constraints such as joint limitations, lumbar pain, and postoperative recovery, while adding safety reminders such as “stop if pain occurs”; as recommended by experts, personalized setting of reminder frequency was added, and Item 1.9 was revised from “fixed reminders every 30 minutes” to “users can set personalized reminder frequencies between 15‐60 minutes”; Item 1.10 supplemented specific interruption methods (eg, standing up and walking) and their rationale on the basis of the original “providing instructions”; to address the unclear demonstration content in Items 1.11 and 3.2, “providing short video cases” was added to enhance demonstration and comprehensibility; In terms of feedback mechanisms: Item 1.12 was adjusted to conduct real-time monitoring through smart wearable devices, generate personalized visual reports, and provide immediate positive feedback after users stand up and move (eg, “Great! You have been active for 5 min, keep it up!").
3. Clarification of social support forms: experts pointed out that the implementation paths of some social support–related items were not clear enough and recommended enhancing interactivity and participation. The research team made the following adjustments: Item 2.1 further clarified the composition of the Question and Answer team to ensure the quality of professional guidance; Items 2.2 and 3.17 were revised from “advising individuals to invite relatives and friends” to “the platform supports users to actively invite relatives and friends, who can receive reminders and send encouraging messages”; Item 2.3 was refined to “the platform matches peers with less sedentary behavior based on user data and supports group challenges” to enhance social interactivity.
4. Stratification and personalization of achievement incentive mechanisms: regarding achievement incentives, experts suggested providing differentiated feedback based on users’ completion status to enhance intervention attractiveness. The research team made corresponding optimizations: Item 3.5 was adjusted from a single “ranking list” to a “progress ranking list,” with encouraging cards such as “Keep going tomorrow” added for users who fail to meet targets; Item 3.6 pushed stratified feedback based on goal achievement: users who complete targets can unlock badges and receive voice congratulations, while those who do not receive encouraging cards; Item 3.11 added expressions such as “you can” and “you will definitely” to make the feedback language more in line with older adults’ usage habits.

#### Intervention Duration

The initial proposed intervention duration was 12 weeks. Experts suggested daily follow-up in the first 2 weeks, with a gradual reduction in frequency thereafter. Additionally, experts recommended adding a “maintenance phase” strategy, converting interventions after 12 weeks to monthly pushes to prevent relapse. Meanwhile, experts noted that behavior change may require a longer period to stabilize. A recent systematic review indicated that significant behavioral changes can be observed with a 12-week intervention, which also has good feasibility. The research team decided to adopt the experts’ suggestions and revised the intervention duration to: “12 weeks (daily follow-up in weeks 1–4 → twice-weekly follow-up in weeks 5–8 → once-weekly consolidation and maintenance in weeks 9–12).”

#### Intervention Forms

The original planned intervention form was a combination of a WeChat (Tencent Holdings Ltd) mini-program and smart wearable devices. However, experts suggested introducing diversified forms such as telephone or remote face-to-face interventions. After discussion, the research team decided to switch to a mobile app, mainly because the app offers stronger functional scalability and a better user experience compared to WeChat mini-programs and can better support diversified forms such as telephone and remote video interventions. Telephone and remote video interventions were integrated into the online consultation module of the app, which not only enhanced intervention flexibility but also avoided overreliance on offline interventions. Meanwhile, experts recommended strengthening the feasibility assessment of smart wearable devices, which will be continuously monitored during subsequent implementation.

#### Indicators for Evaluating Intervention Effects

The originally proposed evaluation indicators included objective sedentary behavior duration, frequency, and count; screen time; activity time; and login time and frequency. Experts suggested adding physiological indicators, subjective indicators (eg, quality of life scale or self-efficacy), and recording “reasons for dropout” to construct a multidimensional evaluation system with subjective-objective cross-validation, incorporating more objective indicators, disease indicators, and satisfaction indicators. Regarding the classification of primary and secondary outcomes of intervention effects, experts recommended clarifying primary and secondary outcomes and noted that the selection of physiological indicators should be consistent with intervention strategies. After 2 rounds of expert consultation, the research team decided to adopt the experts’ suggestions and confirmed the primary outcome indicators as objective sedentary behavior duration, frequency, count, and subjective sedentary behavior (screen time); secondary outcomes include activity time, sedentary behavior, and physical activity–related indicators, physical activity motivation, health literacy, socioeconomic status, social support, waist circumference, weight, blood pressure, quality of life, and feasibility evaluation.

**Figure 2.**
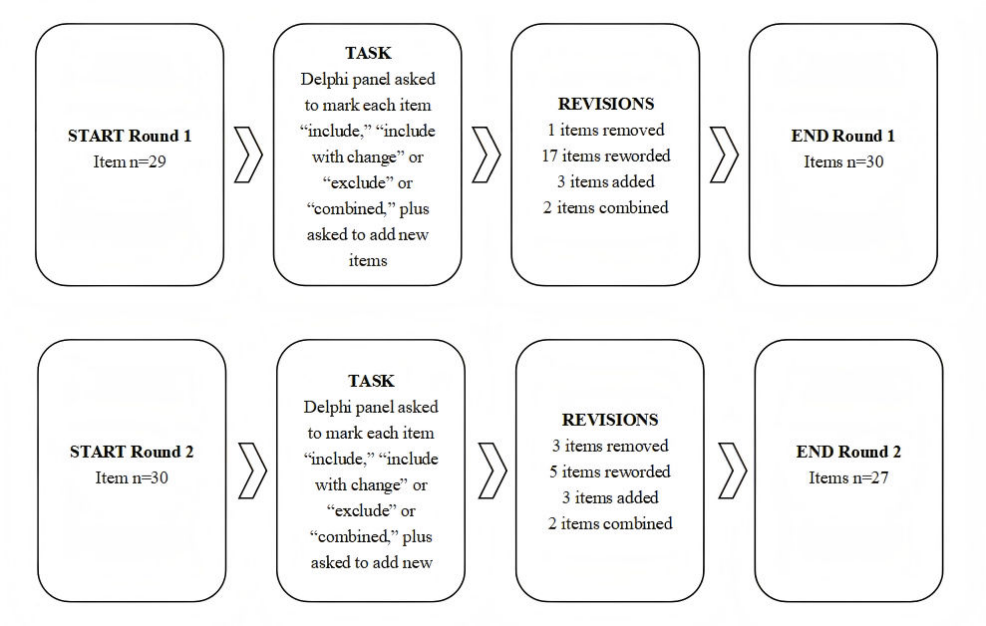
Summary of item modifications across 2 Delphi rounds.

The comprehensive mapping of relationships among the COM-B model, TDF domains, intervention functions, BCTs, and corresponding intervention components, which initially included 29 items, is presented in Multimedia Appendix 3. The expert panel reviewed this initial list and provided feedback, leading to the removal of 1 item, rewording of 17, addition of 3, and combination of 2, resulting in a revised total of 30 items. In the second round, the revised list of 30 items was further reviewed, with additional modifications: 3 items were removed, 5 were reworded, 3 new items were added, and 2 items were combined, yielding a final total of 27 items (Figure 2). A comparison of the preliminary and finalized versions of the mHealth intervention for reducing sedentary behavior in older adults across both rounds is provided in Table 3. A detailed item-by-item comparison of revisions across the 2 rounds is provided in Multimedia Appendix 4, and a comparison of the preliminary and finalized versions in Multimedia Appendix 5.

**Table 3.** Finalized versions of the mobile health intervention for reducing sedentary behavior in older adults^a^.

Intervention element	Intervention (final version)
Intervener	Geriatric and rehabilitation department medical staff, sports science experts, trained researchers, etc
Intervention goal	Reduce older adults’ total sedentary behavior time (≤8 h) and screen time (≤3 h).
Intervention duration	12 weeks (daily follow-up wk 1‐4 → twice-weekly follow-up wk 5‐8 → once-weekly consolidation or maintenance wk 9‐12).
Intervention format	App and wearable devices.
Intervention effect evaluation	Primary outcomes: objective sedentary behavior duration, frequency, count; subjective sedentary behavior (screen time); secondary outcomes: activity time; motivation for sedentary behavior and physical activity; health literacy, socioeconomic status, social support; waist circumference; weight; blood pressure; fasting blood glucose; triglycerides; HDL^b^ cholesterol; total cholesterol; HbA_1c_^c^; quality of life; login time and frequency; dropout reasons; satisfaction.
Intervention content	Provide information via the platform on an overview of sedentary behavior, health risks, and benefits of interrupting sedentary behavior. Reference content from sedentary guidelines of organizations such as WHO^d^ via the platform. Provide information via the platform on methods for interrupting sedentary behavior. Provide information via the platform on sedentary behavior in different scenarios and its health risks, such as watching TV in entertainment scenarios, sedentary office work in work scenarios, and the potential increase in sedentary behavior with age, explaining the adverse health consequences these behaviors may cause. Collaborate with rehabilitation physicians or other professionals to assess physical limitations (eg, restricted joint mobility, lumbar pain, and postoperative recovery), and identify solutions (eg, push customized actions based on health data) to break sedentary behavior, adding safety prompts such as “Stop if pain occurs.” Help individuals accurately and repeatedly practice interrupting sedentary behavior via the platform by triggering a sedentary reminder every 30 minutes, practicing repeatedly until proficient in sedentary interruption skills. Remind users to stand up and interrupt sedentary behavior based on their preferred sedentary interruption frequency setting (15‐60 min) via the platform, synchronously sending notifications such as “You've been sitting for 30 minutes, get up and move!.” Provide information via the platform on methods for interrupting sedentary behavior (eg, standing and walking) and their rationale, and instructions on setting up and using the app. Provide short video exemplary cases via the platform of regularly interrupting sedentary behavior (eg, standing stretch during TV ad breaks, standing activity during long mahjong sessions and getting up for water during long sedentary work), demonstrating how to accurately and correctly interrupt sedentary behavior. The platform monitors user sedentary behavior in real-time (including single-session sedentary duration and daily cumulative sedentary time) via smart wearable devices, automatically generates visual reports, and pushes personalized improvement suggestions. For example, monitoring shows your longest continuous sedentary period was 2 PM-4 PM. Current cumulative sedentary time has reached 7 hours (approaching the 8-h guideline limit). Suggestion: get up and move for 5 minutes immediately; Avoid exceeding 8 hours sedentary time for the rest of today. Automatically provides feedback upon detecting user activity: Great! You have been active for 5 minutes, please keep it up! Push information via the platform on the benefits of interrupting sedentary behavior, emphasizing the necessity for users to receive training. Remind individuals via the platform (eg, during postmeal rest time) to participate in training (eg, “You can learn standing stretches now”). Invite a team including geriatric doctors, rehabilitation therapists, etc, to provide online question-and-answer sessions, offering professional advice and guidance to individuals on reducing sedentary behavior. Users invite family, friends, or colleagues to interrupt sedentary behavior together via the platform, and encourage each other through the platform’s chat function; family members receive reminders and send encouraging messages. The platform intelligently matches users with “partners with low sedentary behavior” based on user behavior data, and supports group challenge competitions (eg, daily cumulative sedentary or standing time PK). Popularize available social support resources (eg, senior activity centers and interest groups) via the platform, explaining the positive role of participating in these social activities in reducing sedentary behavior, guiding them to proactively seek and use related resources. Disseminate information about available environmental resources (eg, community fitness facilities) through the platform, explaining their positive role in reducing sedentary behavior, and guide individuals to proactively access and use these resources. Automatically record the type, time, and frequency of sedentary behavior via wearable devices. Provide videos via the platform featuring health care professionals emphasizing the benefits of interrupting sedentary behavior. Display the user’s progress ranking for sedentary behavior duration within their friend circle via the platform’s “Progress Leaderboard” function, motivating users to reduce sedentary behavior; noncompleters receive a “Try again tomorrow” encouragement card. The platform intelligently pushes layered incentives based on user goal achievement (completers unlock badges such as “Sedentary Slayer” or “Star of Vitality”+voice congratulatory message; noncompleters receive a “Try again tomorrow” encouragement card) and share them on social media to receive likes and encouragement from friends. Require individuals via the platform to use words such as “willing,” “can,” “commit,” or “definitely can,” “high priority” to confirm or reaffirm their commitment to starting, continuing, or restarting attempts to reduce sedentary behavior. Sign a sedentary behavior commitment contract with individuals via the platform to ensure regular interruption of sedentary behavior. Set sedentary behavior reduction goals (eg, “daily sedentary time ≤8 h”) via the platform. Support creation of “Sedentary Behavior Interruption Plans” via the platform, eg, “perform a 5-minute standing stretch at 10:00, 14:00, 16:00 each day,” with reminders via the platform. Generate weekly sedentary behavior data reports, comparing actual sedentary behavior time with goals. Users can view historical records via the platform’s timeline function to assess goal achievement progress. Introduce via the platform how reducing sedentary behavior can alleviate individuals’ negative emotions.

a The final intervention items are derived by merging repeated items from the initial list. Item 1 is derived from Items 1.1 and 1.3; Item 2 is derived from Items 1.2, 1.4, and 3.9; Item 3 is derived from Items 1.6 and 3.3; and Item 4 is derived from Items 1.7 and 3.7. Items 5‐8 are derived from Items 1.8, 1.5, 1.9, and 1.10, respectively. Item 9 is derived from Items 1.11 and 3.2, and Items 10‐12 are derived from Items 1.12‐1.14, respectively. Item 13 is derived from Item 2.1; Item 14 is derived from Items 2.2 and 3.17; Items 15‐18 are derived from Items 2.3, 2.4, 2.6, and 3.1, respectively. Item 19 is derived from Items 3.4 and 3.8; Items 20‐21 are derived from Items 3.5‐3.6, respectively; Items 22‐23 are derived from Items 3.11 and 3.10, respectively; Item 24 is derived from Items 2.5 and 3.12, and Items 25‐27 are derived from Items 3.13, 3.14, and 3.16, respectively. Clinical biomarkers (eg, glycated hemoglobin and lipids) are included as candidate secondary outcomes for future definitive trials; their assessment will depend on feasibility and available resources.

b HDL: high-density lipoprotein.

c HbA_1c_: glycated hemoglobin.

d WHO: World Health Organization.

The figure illustrates the iterative refinement process of the intervention items based on expert feedback in Round 1 and Round 2, showing how the initial 29 items were gradually revised to a final set of 27 items.

**Figure 3.**
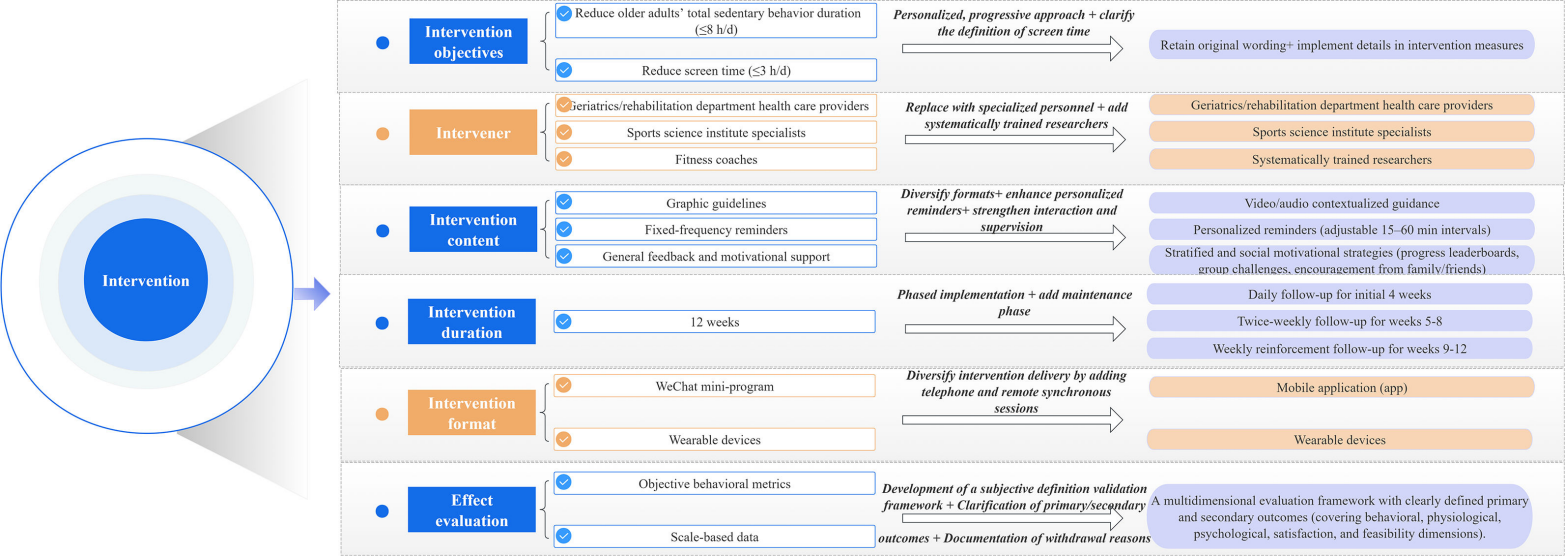
Revised framework of the mobile health intervention for reducing sedentary behavior in older adults.

The revised framework of the mHealth intervention is illustrated in Figure 3.

The framework illustrates the key intervention components, including intervention functions, BCTs, and delivery format, mapped onto the COM-B dimensions.

## Discussion

### Summary of Key Findings

To our knowledge, this study is the first to systematically integrate the BCW framework with the Delphi expert consensus method to develop an mHealth intervention for reducing sedentary behavior in older adults. The final intervention comprises 7 intervention functions and 20 BCTs, operationalized into 27 intervention items. These comprehensively address the 3 core dimensions of the COM-B model: capability, opportunity, and motivation, resulting in a theoretically driven, expert-validated mHealth intervention tailored to the unique needs of older adults. It should be noted that the preliminary development of the mHealth intervention for reducing sedentary behavior in older adults, guided by the 3-phase, 8-step BCW framework, is beyond the scope of this manuscript and will be detailed in a separate publication. This study instead focuses on the Delphi expert consultation process, through which the intervention components were refined and validated by multidisciplinary expert consensus. This validation was achieved through rigorous Delphi procedures with a panel of 16 multidisciplinary experts, all holding a bachelor’s degree or higher (11 with doctoral degrees and 5 with master’s degrees) and 87.5% (14/16) holding associate senior positions or higher, with an average of more than 15 years of professional experience. The 2 Delphi rounds achieved 100% response rates, with expert authority coefficients of 0.900 and 0.907, significantly exceeding the 0.70 threshold, demonstrating strong reliability and stability of expert judgments [13]. Statistically significant concordance (Kendall W=0.151, *P*<.001 and W=0.214, *P*=.001) across both rounds further validates the reliability of the consensus process. Expert feedback across the 2 rounds led to iterative refinements, including item additions, deletions, and content optimization, with suggestions primarily focused on enhancing intervention details and implementation feasibility. Collectively, these findings fulfill the study’s objective of developing a theoretically grounded, expert-validated mHealth intervention using the Delphi method, establishing a robust foundation for subsequent empirical testing.

### Comparison With Existing Literature

Our previous systematic review [6] identified several studies [15-17] that used the Jawbone Up app (Jawbone Inc) to intervene in sedentary behavior among older adults. However, this app was not specifically designed for older adults and has since been discontinued, which may limit its relevance for this population. Moreover, some of these studies [15,18] primarily focused on increasing physical activity to reduce sedentary behavior, rather than directly targeting sedentary behavior itself. As a result, they did not incorporate BCTs specifically intended to reduce sedentary behavior in older adults. Additionally, most of the mobile interventions for sedentary behavior in older adults, including those involving the Jawbone Up App, were pilot studies [15-19]. While they provided preliminary empirical evidence for sedentary behavior reduction, the small sample sizes and certain design limitations raise concerns about the generalizability and reliability of the results, which require further validation. Furthermore, some studies [17,20-22] lacked a systematic foundation in BCTs or behavioral theory, which contributed to suboptimal intervention outcomes.

### Interpretation of Intervention Components Across COM-B Dimensions

In contrast, our mHealth intervention uses a more structured and theoretically grounded design by systematically linking identified barriers to the COM-B model and using this analysis to inform the a priori selection of BCTs. In the intervention design of this study, our research team proposed ultimately adopted 7 intervention functions and 20 specific BCTs, and invited experts to rate each technique on rationality and importance. Based on expert feedback, the principal findings across COM-B or TDF dimensions were as follows: at the level of psychological and physical capability, to address barriers such as older adults’ lack of knowledge about sedentary behavior and limited ability to interrupt it, the research team used the intervention functions of education and enablement together with the BCTs “Information about health consequences” (BCT 5.1), “Credible source” (BCT 9.1), “Prompts or cues” (BCT 7.1), “Problem solving” (BCT 1.2), and “Behavioral practice or rehearsal” (BCT 8.1). Expert ratings indicated that these techniques were generally high on both rationality and importance. For the barrier of lacking methods to interrupt sedentary behavior, the Training function was used with “Instruction on how to perform the behavior” (BCT 4.1), “Demonstration of the behavior” (BCT 6.1), “Behavioral practice or rehearsal” (BCT 8.1), and “Feedback on behavior” (BCT 2.2); experts considered these techniques crucial for building skills to reduce sedentary behavior among older adults. In addition, to mitigate low training priority or limited training time, “Information about health consequences” (BCT 5.1) under Education and “Prompts or cues” (BCT 7.1) also received high endorsement.

At the level of social and physical opportunity, for insufficient social support and environmental constraints, the research team adopted enablement and education, proposing “Social support (unspecified)” (BCT 3.1), “Social support (emotional)” (BCT 3.3), “Information about social and environmental consequences” (BCT 5.3) and “Restructuring the social environment” (BCT 12.2). Expert ratings showed that these techniques were judged highly rational and important for strengthening older adults’ social support and improving the social environment to reduce sedentary behavior.

At the level of reflective and automatic motivation, the research team used modeling, education, persuasion, and incentivization, along with “Self-monitoring of behavior” (BCT 2.3) and “Demonstration of the behavior” (BCT 6.1) to help older adults integrate reduced sedentary behavior into their role identity and enhance their confidence in changing their behavior. In addition, to explicitly strengthen older adults’ intentions and goal-setting for behavior change, the research team used “Credible source” (BCT 9.1), “Commitment” (BCT 1.9), “Social reward” (BCT 10.4), and “Goal setting (behavior)” (BCT 1.1). These techniques received high expert endorsements for both rationality and importance. For automatic motivation, incentivization was adopted, and experts particularly highlighted “Social reward” (BCT 10.4), “Information about emotional consequences” (BCT 5.6), and “Social support (emotional)” (BCT 3.3) as effective for eliciting positive emotional experiences and intrinsic motivation to reduce sedentary behavior among older adults. Overall, the BCTs selected by the research team received high ratings for rationality and importance.

### Significance and Innovations

The practical implications of this intervention are manifold, featuring 3 key innovations: intelligent monitoring with real-time feedback for precise behavior modification, a multitiered social support system encompassing professional guidance and family involvement, and motivational architectures combining gamification elements with goal-setting strategies. These components collectively address the 3 essential conditions for behavior change—capability, opportunity, and motivation—as outlined in the COM-B model, providing a comprehensive approach rarely seen in existing interventions for older adults. The intervention’s design specifically tackles implementation challenges through features such as family engagement tools and culturally adapted activity suggestions, which enhance both feasibility and sustainability. This represents a promising step from traditional, clinic-based interventions to more sustainable, technology-enabled community-embedded approaches that align with modern health care delivery models.

Moreover, this study developed an mHealth intervention focusing on the 3 core dimensions of capability, opportunity, and motivation. The intervention’s unique approach lies in its comprehensive and targeted strategy, which not only enhances older adults’ knowledge and skills but also provides appropriate environmental support and strengthens motivation for behavior change. Through 2 rounds of expert feedback and optimization, the intervention content was continuously refined to ensure its effectiveness and feasibility. In terms of capability, the intervention uniquely improves older adults’ awareness of sedentary behavior through educational content while also providing practical guidance to help them master specific behaviors to interrupt sedentary activity. The program educates users about the health risks of prolonged sedentary behavior, such as cardiovascular diseases, metabolic disorders, and cognitive decline, motivating them to change their behavior. Additionally, the intervention offers clear, step-by-step instructions on how to break sedentary behavior, including standing, walking, and stretching. The intervention also includes a flexible sedentary interruption reminder system, allowing users to customize the frequency and timing of reminders to meet their individual needs, improving the intervention’s adaptability and feasibility. Expert feedback in the first round focused on improving the intervention’s operability and clarity, with suggestions to convert text-based materials into video demonstrations with voice guidance, significantly improving accessibility. In the second round, experts recommended incorporating real-time behavioral feedback via wearable devices, helping users detect and adjust their sedentary behavior, thereby enhancing the intervention’s real-time and personalized nature. Regarding opportunity, the intervention creates a supportive environment by promoting both social support and optimizing the physical environment. It encourages older adults to involve family, friends, or colleagues, thus increasing social support and overcoming feelings of isolation and lack of motivation. Feedback from the experts in the first round emphasized enhancing social support by encouraging older adults to invite family, friends, or colleagues to participate, which would foster a sense of community. In the second round, experts suggested adding features such as “sedentary duration challenges” to promote friendly competition, further boosting engagement and long-term adherence. In terms of motivation, the intervention combines intrinsic and extrinsic motivation strategies, including personalized goal-setting, rewards, and social sharing, to encourage continuous behavior change. It guides older adults to set specific goals, such as reducing daily sedentary time, and encourages them to share their progress on social platforms, involving family and friends. Additionally, external motivation is provided through achievement badges and leaderboards, motivating users to push themselves and overcome barriers to behavior change. Expert feedback on motivation focused on enhancing personalization and social interaction. In the first round, experts suggested showcasing user progress and incorporating social incentives, such as progress leaderboards and social rewards. The second round further optimized this mechanism by introducing encouragement cards for users who did not meet their goals and unlocking additional rewards for those who achieved them.

The innovation of this study lies in its comprehensive, multidimensional approach to reducing sedentary behavior in older adults. By integrating the BCW framework, the intervention uniquely addresses capability, opportunity, and motivation to create a personalized and adaptable solution for behavior change. It combines knowledge-building, social support, and real-time feedback, allowing older adults to understand the health risks of sedentary behavior and actively engage in strategies to interrupt it. The use of wearable devices for monitoring and providing personalized feedback adds a real-time, data-driven element to the intervention, enhancing its relevance and effectiveness. Additionally, the focus on social interaction features such as “sedentary duration challenges” promotes long-term engagement and adherence. The inclusion of motivational strategies, such as personalized goal-setting, achievement badges, and social rewards, strengthens both intrinsic and extrinsic motivation, ensuring continued participation and behavior change. Future research should focus on conducting large-scale randomized controlled trials to rigorously assess the long-term efficacy of the intervention in real-world settings, refining age-appropriate design elements to improve usability for older adults, and exploring how technology can be further tailored to meet the diverse physical and cognitive needs of this population.

### Limitations

This study has several limitations. First, although the Delphi panel included experts from multiple relevant disciplines, the representativeness of the expert panel may be limited, and the perspectives obtained may not fully reflect all populations involved in sedentary behavior interventions for older adults. Second, this study focused on the development of the mHealth intervention through expert consensus and did not include end user involvement or empirical evaluation at this stage. Future research is needed to pilot test the intervention and examine its feasibility, usability, and effectiveness through empirical studies, such as randomized controlled trials.

### Conclusion

This study, grounded in the BCW framework, systematically integrates mixed qualitative and quantitative research methods, mHealth technology, and 2 rounds of expert consultation to develop an mHealth intervention targeting sedentary behavior in older adults. Comprising 27 intervention items, the intervention includes key elements such as goals, content, duration, and delivery format, focusing on enhancing older adults’ capabilities, creating opportunities, and motivating behavior change. This intervention has the potential to address the dual needs of older adults and health care providers in managing sedentary behavior. Future research should focus on validating its effectiveness, optimizing its application, and assessing its scalability across diverse populations to maximize its impact.

## Supplementary material

10.2196/83302 Multimedia Appendix 1 Demographic characteristics of the research team and consulting experts.

10.2196/83302 Multimedia Appendix 2 Expert consultation feedback and revisions in 2 rounds.

10.2196/83302 Multimedia Appendix 3 Mapping of Capability, Opportunity, Motivation–Behavior model, Theoretical Domains Framework, intervention functions, behavior change techniques, and sedentary behavior intervention content with expert consultation results.

10.2196/83302 Multimedia Appendix 4 Comparison of item revisions in 2 rounds.

10.2196/83302 Multimedia Appendix 5 Comparison of preliminary and finalized versions of the mHealth intervention for reducing sedentary behavior in older adults in 2 rounds.

10.2196/83302 Checklist 1 CREDES guidelines.

## Abbreviations

APEASE: Affordability, Practicability, Effectiveness and cost-effectiveness, Acceptability, Side-effects/safety, and Equity
BCT: behavior change technique
BCW: Behavior Change Wheel
COM-B: Capability, Opportunity, Motivation–Behavior
CREDES: Conducting and Reporting of Delphi Studies
CV: coefficient of variation
mHealth: mobile health
TDF: Theoretical Domains Framework
WHO: World Health Organization
