# Supplementary material for: Development of an mHealth Intervention for Reducing Sedentary Behavior in Older Adults: Delphi Study
Source: J Med Internet Res. 2026 Jun 11;28:e83302. doi: 10.2196/83302 (PMC13256482; doi:10.2196/83302)
Supplement: Multimedia Appendix 1 [file jmir-v28-e83302-s001.docx]

**Multimedia Appendix 1**

**Table S1.** Demographic characteristics of research team members.

| **ID** | **Highest Degree** | **Professional Title** | **Expertise/Specialization** |
| --- | --- | --- | --- |
| 1 | PhD | Chief Nurse Specialist | Cardiovascular health and sedentary behavior |
| 2 | PhD | Professor | Exercise physiology |
| 3 | PhD | Associate Professor | Psychological stress, motivation and sedentary behavior |
| 4 | PhD | Research Fellow | Physical activity, sedentary behavior and sitting physiology |
| 5 | PhD | Research Fellow | Physical activity and health |
| 6 | PhD candidate | Nurse-in-charge | Cardiovascular health and sedentary behavior |
| 7 | Master's | Nurse | Cardiovascular health and sedentary behavior |

**Table S2.** Demographic characteristics of consulting experts.

| ID | Sex | Age (years) | Work experience (years) | | Education | Professional title | Position | Specialty |
| --- | --- | --- | --- | --- | --- | --- | --- | --- |
| 1 | F | 33 | 2 | PhD | | Assistant Professor | Lecturer | Chronic disease management |
| 2 | F | 45 | 21 | Master | | Chief Nurse | Head Nurse | Cardiovascular rehabilitation |
| 3 | F | 40 | 28 | Master | | Associate Chief Nurse | Clinical nurse | Clinical medicine |
| 4 | F | 46 | 28 | PhD | | Associate Professor | Master's supervisor | Geriatric nursing |
| 5 | F | 60 | 40 | PhD | | Chief Nurse | Doctoral supervisor | Clinical nursing |
| 6 | M | 56 | 34 | PhD | | Professor | Doctoral supervisor | Sports and exercise science |
| 7 | F | 56 | 38 | PhD | | Professor | Lecturer | Geriatric nursing |
| 8 | F | 43 | 20 | Master | | Associate Chief Nurse | Head Nurse | Nursing education |
| 9 | F | 31 | 5 | PhD | | Associate Researcher | Lecturer | Health and chronic disease management |
| 10 | F | 34 | 12 | Master | | Nurse-in-charge | Head Nurse | Chronic disease management |
| 11 | F | 40 | 12 | PhD | | Professor | Doctoral supervisor | Exercise and health promotion |
| 12 | F | 27 | 1 | PhD | | Assistant Professor | None | Health behavior intervention |
| 13 | F | 41 | 14 | PhD | | Professor | Vice Dean | Clinical nursing |
| 14 | F | 35 | 7 | PhD | | Researcher | Associate Director of Nursing Department | Geriatric health and chronic disease management |
| 15 | F | 41 | 18 | Master | | Associate Chief Nurse | Assistant Director of Nursing Department | Chronic disease management |
| 16 | F | 38 | 6 | PhD | | Researcher | Doctoral supervisor | Exercise physiology  and health promotion |

Note: F=female; M=male
