# Supplementary material for: Development of an mHealth Intervention for Reducing Sedentary Behavior in Older Adults: Delphi Study
Source: J Med Internet Res. 2026 Jun 11;28:e83302. doi: 10.2196/83302 (PMC13256482; doi:10.2196/83302)
Supplement: Multimedia Appendix 3 [file jmir-v28-e83302-s003.docx]

**Multimedia Appendix 3**

**Table S1.** Mapping of COM-B Model, TDF, Intervention Functions, BCTs, and Sedentary Behavior Intervention Content.

| **COM-B** | **TDF** | **Barrier Factors** | | **Intervention Function** | **BCTs** | **Intervention Content** |
| --- | --- | --- | --- | --- | --- | --- |
|  |  |  |  |  |  |  |
| Psychological Capability | Knowledge | Lack of knowledge about sedentary behavior | | Education | 5.1 Information about health consequences | Provide information via the platform on the definition of sedentary behavior, health risks, and benefits of interrupting sedentary behavior. |
|  | Behavioral Regulation | Lack of methods knowledge to interrupt sedentary behavior | | Training | 4.1 Instruction on how to perform the behavior | Provide instructions via the platform on how to interrupt sedentary behavior (e.g. standing, walking). |
|  |  |  |  |  | 6.1 Demonstration of the behavior | Provide a "Sedentary Interruption Guide" via the platform, including illustrated/text tutorials for 10 simple stretching exercises (e.g. "Standing Leg Stretch"), users can learn directly within the app. |
|  |  |  |  |  | 8.1 Behavioral practice/rehearsal | Encourage individuals via the platform to accurately and repeatedly practice interrupting sedentary behavior. |
|  |  |  |  | Education | 5.1 Information about health consequences | Provide information via the platform on methods for interrupting sedentary behavior. |
|  | Memory, Attention and Decision Processes | Religious activities promote sedentary behavior | | Education | 5.1 Information about health consequences | Provide information via the platform on the adverse health consequences of sedentary behavior and the benefits of interrupting it. |
|  |  | Entertainment activities promote sedentary behavior | |  |  |  |
|  |  | Work demands promote sedentary behavior | |  |  |  |
|  |  | Aging promotes sedentary behavior | |  |  |  |
|  |  | Physical limitations promote sedentary behavior | | Enablement | 1.2 Problem solving | Collaborate with health coaches or other professionals to assess barriers (e.g. physical limitations) and identify solutions to break sedentary behavior. |
|  |  | Subjective sedentary habits promote sedentary behavior | |  | 8.1 Behavioral practice/rehearsal | Encourage individuals via the platform to accurately and repeatedly practice interrupting sedentary behavior. |
|  |  | Lack of knowledge about sedentary behavior | | Education | 5.1 Information about health consequences | Provide information via the platform on the adverse health consequences of sedentary behavior and the benefits of interrupting it. |
|  |  | No program or reminders for sedentary behavior | | Environmental Restructuring | 7.1 Prompts/cues | Remind users via the platform at preset times (e.g.every 30 minutes) to stand up and interrupt sedentary behavior, synchronously sending a notification "You've been sitting for 30 minutes, get up and move!". |
| Physical Capability | Skills | Lack of understanding of skills to manage sedentary behavior | | Training | 4.1 Instruction on how to perform the behavior | Provide instructions via the platform on how to interrupt sedentary behavior and on setting up and using the application. |
|  |  | Limited understanding of skills to manage sedentary behavior | |  | 6.1 Demonstration of the behavior | Provide exemplary cases via the platform of regularly interrupting sedentary behavior, to demonstrate how to accurately and correctly interrupt sedentary behavior. |
|  |  | Lack of methods to interrupt sedentary behavior | |  | 8.1 Behavioral practice/rehearsal | Encourage individuals via the platform to accurately and repeatedly practice interrupting sedentary behavior. |
|  |  |  |  |  | 2.2 Feedback on behavior | Provide feedback via the platform on areas for improvement in the individual's interruption of sedentary behavior. |
|  |  | Underprioritization of sedentary behavior reduction skills | | Education | 5.1 Information about health consequences | Provide information via the platform on the adverse health consequences of sedentary behavior and the benefits of interrupting it. |
|  |  | Believes training is unnecessary | |  |  | Push information via the platform on the benefits of interrupting sedentary behavior, emphasizing the necessity for older adults to receive training. |
|  |  | Lack of time for training | | Environmental Restructuring | 7.1 Prompts/cues | Remind individuals via the platform to arrange time reasonably to participate in training to reduce sedentary behavior, e.g. suggest individuals reduce entertainment time to decrease sedentary behavior. |
| Social Opportunity | Social Influences | Lack of social support | | Enablement | 3.1 Social support (unspecified) | Invite a team including doctors, rehabilitation therapists, fitness coaches, etc., to provide online question-and-answer sessions, offering professional advice and guidance to individuals on reducing sedentary behavior. |
|  |  |  |  |  | 3.3 Social support (emotional) | Suggest via the platform that individuals invite family, friends, or colleagues to interrupt sedentary behavior together, and encourage each other through the platform's chat function. |
|  |  |  |  |  | 12.2 Restructuring the social environment | Suggest via the platform that individuals make friends with people who have low sedentary behavior. |
| Physical Opportunity | Environmental Context/Resources | Environmental or condition limitations | |  | 3.1 Social support (unspecified) | Invite a team including doctors, rehabilitation therapists, fitness coaches, etc., to provide online question-and-answer sessions, offering professional advice and guidance to individuals on reducing sedentary behavior. |
|  |  | Family environment affects sedentary behavior | |  |  |  |
|  |  | Work limitations | |  | 1.1 Goal setting (behavior) | Set sedentary behavior reduction goals (e.g. "daily sedentary time ≤ 8 hours") via the platform. |
| Reflective Motivation | Social/Professional Role/Identity | Sedentary behavior has not become part of the role | | Modelling | 2.3 Self-monitoring of behavior | Automatically record the type, time, and frequency of sedentary behavior via the platform's built-in accelerometer, generating an hourly sedentary heatmap, allowing users to view details like "Most sedentary between 2-4 PM". |
|  |  |  |  |  | 6.1 Demonstration of the behavior | Provide exemplary cases via the platform of regularly interrupting sedentary behavior. |
|  |  |  |  | Education | 5.1 Information about health consequences | Push information via the platform on the benefits of interrupting sedentary behavior. |
|  | Beliefs about Capabilities | Unclear | | Persuasion | 9.1 Credible source | Provide videos via the platform featuring healthcare professionals emphasizing the benefits of interrupting sedentary behavior. |
|  |  | indifferent or resigned attitude towards the status quo | |  |  |  |
|  |  | Lack of confidence | | Incentivisation | 6.2 Social comparison | Display the user's ranking of sedentary behavior duration within their friend circle via the platform's "Leaderboard" function, motivating users to reduce sedentary behavior to improve their ranking. |
|  |  | Confidence, but lack of motivation | |  | 10.4 Social reward | After achieving sedentary behavior reduction goals, users can unlock achievement badges (e.g. "Sedentary Slayer") and share them on social media to receive likes and encouragement from friends. |
|  |  | half-confidence | |  |  |  |
|  | Beliefs about Consequences | Uncertain about the consequences of sedentary behavior | | Education | 5.1 Information about health consequences | Provide information via the platform on the adverse health consequences of sedentary behavior and the benefits of interrupting it. |
|  | Optimism | Confidence in positive results from reducing sedentary behavior (Low) | | Persuasion |  | On the platform, introduce the benefits of interrupting sedentary behavior through videos or health consultations. |
|  |  |  |  |  | 9.1 Credible source | Reference content from sedentary guidelines of organizations like WHO (e.g. "at least 1 minute of activity per hour") in the platform's sedentary classroom module to enhance user trust. |
|  | Intentions | Unwillingness to interrupt sedentary behavior | |  | 5.1 Information about health consequences | On the platform, introduce the benefits of interrupting sedentary behavior through videos or health consultations. |
|  |  |  |  |  | 9.1 Credible source | Provide videos via the platform featuring healthcare professionals emphasizing the benefits of interrupting sedentary behavior. |
|  |  |  |  | Modelling | 1.8 Behavioral contract | Sign a sedentary commitment contract with individuals via the platform to ensure regular interruption of sedentary behavior. |
|  |  |  |  |  | 1.9 Commitment | Require individuals via the platform to use words such as "strongly," "commit," or "high priority," "I will" to confirm or reaffirm their commitment to starting, continuing, or restarting attempts to reduce sedentary behavior. |
|  |  |  |  |  | 6.1 Demonstration of the behavior | Provide exemplary cases via the platform of regularly interrupting sedentary behavior. |
|  | Goals | No clear goal | | Education | 5.1 Information about health consequences | On the platform, introduce the benefits of interrupting sedentary behavior through videos or health consultations. |
|  |  |  |  | Enablement | 1.1 Goal setting (behavior) | Set sedentary behavior reduction goals (e.g. "Daily sedentary time ≤ 8 hours") via the platform. |
|  |  |  |  |  | 1.4 Action planning | Support creation of "Sedentary Interruption Plans" via the platform, e.g. "Perform a 5-minute standing stretch at 10:00, 14:00, 16:00 each day", with reminders via the platform. |
|  |  |  |  |  | 1.5 Review behavior goal(s) | Generate weekly sedentary data reports, comparing actual sedentary time with goals. Users can view historical records via the platform's timeline function to assess goal achievement progress. |
|  |  |  |  | Incentivisation | 10.4 Social reward | After achieving sedentary behavior reduction goals, users can unlock achievement badges (e.g. "Sedentary Slayer") and share them on social media to receive likes and encouragement from friends. |
| Automatic Motivation | Reinforcing Behavior | Lack of motivation | |  | 5.1 Information about health consequences | Provide specific information via the platform on the health risks of sedentary behavior (e.g. "Each additional hour of sedentary time increases diabetes risk by 9%"). |
|  |  |  |  |  | 10.4 Social reward | After achieving sedentary behavior reduction goals, users can unlock achievement badges (e.g. "Sedentary Slayer") and share them on social media to receive likes and encouragement from friends. |
|  | Emotion | No emotional change | |  | 5.6 Information about emotional consequences | Introduce via the platform how reducing sedentary behavior can alleviate individuals' negative emotions. |
|  |  |  |  |  | 3.3 Social support (emotional) | Suggest via the platform that individuals invite family, friends, or colleagues to interrupt sedentary behavior together, and encourage each other through the app's chat function. |
| Intervener | | | Geriatric, rehabilitation department medical staff, sports science experts, fitness coaches, etc. | | | |
| Intervention Goal | | | Reduce older adults' total sedentary behavior time (≤8 hours) and screen time (≤3 hours). Note: Referencing the Canadian 24-Hour Movement Guidelines. | | | |
| Intervention Duration | | | 12 weeks | | | |
| Intervention Format | | | WeChat Mini Program and Metawear wearable device | | | |
| Intervention Effect Evaluation | | | Objective sedentary behavior duration, frequency, count; Screen time; Activity time; Login time and frequency, etc. | | | |

**Table S2.** Results of Expert Consultation on mHealth Intervention for Changing Sedentary Behavior in Older Adults (Intervention Functions and BCTs).

| **COM-B** | **TDF** | **Barrier Factors** | **Intervention Function** | **BCTs** | **Rationality (Mean±SD, points)** | **Importance (Mean±SD, points)** | **CV** |
| --- | --- | --- | --- | --- | --- | --- | --- |
| Psychological Capability | Knowledge | Lack of related Knowledge About Sedentary Behavior | Education | 5.1 Information about health consequences | 4.94±0.25 | 4.94±0.25 | 0.05 |
|  |  |  |  | 9.1 Credible source | 4.69±0.70 | 4.75±0.58 | 0.12 |
|  | Behavioral Regulation | Lack of knowledge on how to interrupt sedentary behavior | Education | 5.1 Information about health consequences | 4.56±0.73 | 4.50±0.82 | 0.18 |
|  | Memory, Attention and Decision Processes | Entertainment activities promote sedentary behavior | Education | 5.1 Information about health consequences | 4.75±0.58 | 4.75±0.58 | 0.12 |
|  |  | Work demands promote sedentary behavior |  |  |  |  |  |
|  |  | Aging promotes sedentary behavior |  |  |  |  |  |
|  |  | Physical limitations promote sedentary behavior | Enablement | 1.2 Problem solving | 4.88±0.34 | 4.88±0.34 | 0.07 |
|  |  | Habit-driven sedentary behavior |  | 8.1 Behavioral practice/rehearsal | 4.94±0.25 | 4.94±0.25 | 0.05 |
|  |  | Absence of behavioral change program prompts | Environmental Restructuring | 7.1 Prompts/cues | 5.00±0.00 | 5.00±0.00 | 0.00 |
| Physical Capability | Skills | Lack of methods to reduce sedentary behavior | Training | 4.1 Instruction on how to perform the behavior | 5.00±0.00 | 5.00±0.00 | 0.00 |
|  |  |  |  | 6.1 Demonstration of the behavior | 5.00±0.00 | 5.00±0.00 | 0.00 |
|  |  |  |  | 8.1 Behavioral practice/rehearsal | 5.00±0.00 | 5.00±0.00 | 0.00 |
|  |  |  |  | 2.2 Feedback on behavior | 5.00±0.00 | 5.00±0.00 | 0.00 |
|  |  | Underprioritization of sedentary behavior reduction skills | Education | 5.1 Information about health consequences | 4.69±0.60 | 4.69±0.60 | 0.13 |
|  |  | Believes training is unnecessary |  |  |  |  |  |
|  |  | Lack of time for training | Environmental Restructuring | 7.1 Prompts/cues | 4.69±0.70 | 4.69±0.70 | 0.15 |
| Social Opportunity | Social Influences | Lack of social support | Enablement | 3.1 Social support (unspecified) | 5.00±0.00 | 4.88±0.34 | 0.07 |
|  |  |  |  | 3.3 Social support (emotional) | 4.81±0.54 | 5.00±0.00 | 0.00 |
|  |  |  |  | 12.2 Restructuring the social environment | 5.00±0.00 | 4.88±0.34 | 0.07 |
|  |  |  | Education | 5.3 Information about social and environmental consequences | 4.69±0.60 | 4.88±0.34 | 0.07 |
| Physical Opportunity | Environmental Context/Resources | Environmental or condition limitations | Enablement | 3.1 Social support (unspecified) | 4.94±0.25 | 4.94±0.25 | 0.05 |
|  |  |  |  | 1.1 Goal setting (behavior) | 4.69±0.70 | 4.69±0.70 | 0.15 |
|  |  |  | Education | 5.3 Information about social and environmental consequences | 4.88±0.34 | 5.00±0.00 | 0.00 |
| Reflective Motivation | Social/Professional Role/Identity | Sedentary behavior has not become part of the role | Modelling | 2.3 Self-monitoring of behavior | 4.94±0.25 | 4.94±0.25 | 0.05 |
|  |  |  |  | 6.1 Demonstration of the behavior | 4.81±0.54 | 4.94±0.25 | 0.05 |
|  |  |  | Education | 5.1 Information about health consequences | 4.94±0.25 | 4.94±0.25 | 0.05 |
|  | Beliefs about Capabilities | Ambivalence regarding self-confidence | Persuasion | 9.1 Credible source | 4.94±0.25 | 4.94±0.25 | 0.05 |
|  |  | Indifferent or resigned attitude towards the status quo |  | 6.2 Social comparison | 4.88±0.34 | 4.88±0.34 | 0.07 |
|  |  | Lack of confidence | Incentivisation | 10.4 Social reward | 4.94±0.25 | 4.94±0.25 | 0.05 |
|  |  | Confidence, but lack of motivation |  |  |  |  |  |
|  | Beliefs about Consequences | Uncertain about the consequences of sedentary behavior | Education | 5.1 Information about health consequences | 5.00±0.00 | 4.94±0.25 | 0.05 |
|  | Optimism | Confidence in Positive Results from Improving Sedentary Behavior (Low) | Persuasion | 5.1 Information about health consequences | 4.94±0.25 | 4.94±0.25 | 0.05 |
|  |  |  |  | 9.1 Credible source | 4.94±0.25 | 4.94±0.25 | 0.05 |
|  | Intentions | Unwillingness to Reduce Sedentary Behavior | Persuasion | 5.1 Information about health consequences | 4.88±0.34 | 4.88±0.34 | 0.07 |
|  |  |  |  | 9.1 Credible source | 4.75±0.58 | 4.75±0.58 | 0.12 |
|  |  |  | Modelling | 1.8 Behavioral contract | 4.88±0.34 | 4.81±0.40 | 0.08 |
|  |  |  |  | 1.9 Commitment | 4.94±0.25 | 4.88±0.34 | 0.07 |
|  |  |  |  | 6.1 Demonstration of the behavior | 4.88±0.34 | 4.81±0.54 | 0.11 |
|  |  |  | Incentivisation | 10.4 Social reward | 5.00±0.00 | 5.00±0.00 | 0.00 |
|  | Goals | No Clear Goal | Education | 5.1 Information about health consequences | 4.69±0.70 | 4.88±0.34 | 0.07 |
|  |  |  | Enablement | 1.1 Goal setting (behavior) | 5.00±0.00 | 5.00±0.00 | 0.00 |
|  |  |  |  | 1.4 Action planning | 4.94±0.25 | 4.94±0.25 | 0.05 |
|  |  |  |  | 1.5 Review behavior goal(s) | 4.94±0.25 | 4.94±0.25 | 0.05 |
|  |  |  | Incentivisation | 10.4 Social reward | 5.00±0.00 | 4.94±0.25 | 0.05 |
| Automatic Motivation | Reinforcing Behavior | Lack of Motivation | Incentivisation | 5.1 Information about health consequences | 4.81±0.40 | 4.75±0.45 | 0.09 |
|  |  |  |  | 10.4 Social reward | 4.94±0.25 | 4.81±0.40 | 0.08 |
|  | Emotion | No Emotional Change |  | 5.6 Information about emotional consequences | 4.94±0.25 | 5.00±0.00 | 0.00 |
|  |  |  |  | 3.3 Social support (emotional) | 5.00±0.00 | 4.88±0.34 | 0.07 |

Note: CV, coefficient of variation; SD, standard deviation.

**Table S3.** Results of Expert Consultation on Mobile Health Intervention Programs for Changing Sedentary Behavior in Older Adults (mHealth Intervention).

| **Item** | **Rationality (Mean±SD, points)** | **Importance (Mean±SD, points)** | **CV** |
| --- | --- | --- | --- |
| 1. Provide information via the platform on an overview of sedentary behavior, health risks, and benefits of interrupting sedentary behavior. | 5.00±0.00 | 5.00±0.00 | 0.00 |
| 2. Reference content from sedentary guidelines of organizations like WHO via the platform. | 4.78±0.67 | 5.00±0.00 | 0.00 |
| 3. Provide information via the platform on methods for interrupting sedentary behavior. | 4.44±0.73 | 4.40±0.91 | 0.21 |
| 4. Provide information via the platform on sedentary behavior in different scenarios and its health risks, such as watching TV in entertainment scenarios, sedentary office work in work scenarios, and the potential increase in sedentary behavior with age, explaining the adverse health consequences these behaviors may cause. | 4.67±0.50 | 4.78±0.44 | 0.09 |
| 5. Collaborate with rehabilitation physicians or other professionals to assess physical limitations (e.g. restricted joint mobility, lumbar pain, post-operative recovery), and identify solutions (e.g. push customized actions based on health data) to break sedentary behavior, adding safety prompts such as "Stop if pain occurs". | 4.67±0.50 | 4.67±0.50 | 0.11 |
| 6. Help individuals accurately and repeatedly practice interrupting sedentary behavior via the platform by triggering a sedentary reminder every 30 minutes, practicing repeatedly until proficient in sedentary interruption skills. | 4.89±0.33 | 4.78±0.44 | 0.09 |
| 7. Remind users to stand up and interrupt sedentary behavior based on their preferred sedentary interruption frequency setting (15-60 minutes) via the platform, synchronously sending notifications like "You've been sitting for 30 minutes, get up and move!". | 4.89±0.33 | 4.89±0.33 | 0.07 |
| 8. Provide information via the platform on methods for interrupting sedentary behavior (e.g. standing, walking) and their rationale, and instructions on setting up and using the application. | 5.00±0.00 | 5.00±0.00 | 0.00 |
| 9. Provide short video exemplary cases via the platform of regularly interrupting sedentary behavior (e.g. standing stretch during TV ad breaks; standing activity during long mahjong sessions; getting up for water during long sedentary work), demonstrating how to accurately and correctly interrupt sedentary behavior. | 4.89±0.33 | 4.89±0.33 | 0.07 |
| 10. The platform monitors user sedentary behavior in real-time (including single-session sedentary duration and daily cumulative sedentary time) via smart wearable devices, automatically generates visual reports, and pushes personalized improvement suggestions. E.g. Monitoring shows your longest continuous sedentary period was 14:00-16:00. Current cumulative sedentary time has reached 7 hours (approaching the 8-hour guideline limit). Suggestion: Get up and move for 5 minutes immediately; Avoid exceeding 8 hours sedentary time for the rest of today. Automatically provides feedback upon detecting user activity: Great! You have been active for 5 minutes, please keep it up! | 5.00±0.00 | 5.00±0.00 | 0.00 |
| 11. Push information via the platform on the benefits of interrupting sedentary behavior, emphasizing the necessity for users to receive training. | 5.00±0.00 | 5.00±0.00 | 0.00 |
| 12. Remind individuals via the platform (e.g. during post-meal rest time) to participate in training (e.g."You can learn standing stretches now"). | 4.78±0.44 | 5.00±0.00 | 0.00 |
| 13. Invite a team including geriatric doctors, rehabilitation therapists, etc., to provide online question-and-answer sessions, offering professional advice and guidance to individuals on reducing sedentary behavior. | 4.67±0.50 | 4.56±0.53 | 0.12 |
| 14. Users invite family, friends, or colleagues to interrupt sedentary behavior together via the platform, and encourage each other through the platform's chat function; family members receive reminders and send encouraging messages. | 4.78±0.44 | 4.56±0.73 | 0.16 |
| 15. The platform intelligently matches users with "partners with low sedentary behavior" based on user behavior data, and supports group challenge competitions (e.g. daily cumulative sedentary/standing time PK). | 4.67±0.50 | 4.44±0.73 | 0.16 |
| 16. Popularize available social support resources (e.g. senior activity centers, interest groups) via the platform, explaining the positive role of participating in these social activities in reducing sedentary behavior, guiding them to proactively seek and utilize related resources. | 4.89±0.33 | 4.78±0.44 | 0.09 |
| 17. Disseminate information about available environmental resources (e.g. community fitness facilities) through the platform, explaining their positive role in reducing sedentary behavior, and guide individuals to proactively access and utilize these resources. | 4.89±0.33 | 4.78±0.44 | 0.09 |
| 18. Automatically record the type, time, and frequency of sedentary behavior via wearable devices. | 4.67±0.50 | 4.89±0.33 | 0.07 |
| 19. Provide videos via the platform featuring healthcare professionals emphasizing the benefits of interrupting sedentary behavior. | 4.89±0.33 | 5.00±0.00 | 0.00 |
| 20. Display the user's progress ranking for sedentary behavior duration within their friend circle via the platform's "Progress Leaderboard" function, motivating users to reduce sedentary behavior; non-completers receive a "Try again tomorrow" encouragement card. | 4.78±0.44 | 4.78±0.44 | 0.09 |
| 21. The platform intelligently pushes layered incentives based on user goal achievement (completers unlock badges like "Sedentary Slayer" or "Star of Vitality" + voice congratulatory message; non-completers receive a "Try again tomorrow" encouragement card) and share them on social media to receive likes and encouragement from friends. | 4.78±0.44 | 4.78±0.44 | 0.09 |
| 22. Require individuals via the platform to use words such as "willing," "can," "commit," or "definitely can," "high priority" to confirm or reaffirm their commitment to starting, continuing, or restarting attempts to reduce sedentary behavior. | 4.56±0.53 | 4.44±0.73 | 0.16 |
| 23. Sign a sedentary behavior commitment contract with individuals via the platform to ensure regular interruption of sedentary behavior. | 4.44±0.73 | 4.44±0.88 | 0.20 |
| 24. Set sedentary behavior reduction goals (e.g. "daily sedentary time ≤ 8 hours") via the platform. | 4.89±0.33 | 4.89±0.33 | 0.07 |
| 25. Support creation of "Sedentary Behavior Interruption Plans" via the platform, e.g. "perform a 5-minute standing stretch at 10:00, 14:00, 16:00 each day", with reminders via the platform. | 4.89±0.33 | 5.00±0.00 | 0.00 |
| 26. Generate weekly sedentary behavior data reports, comparing actual sedentary behavior time with goals. Users can view historical records via the platform's timeline function to assess goal achievement progress. | 5.00±0.00 | 5.00±0.00 | 0.00 |
| 27. Introduce via the platform how reducing sedentary behavior can alleviate individuals' negative emotions. | 4.67±0.50 | 4.67±0.50 | 0.11 |

Note: CV, coefficient of variation; SD, standard deviation.
