# Supplementary material for: Development of an mHealth Intervention for Reducing Sedentary Behavior in Older Adults: Delphi Study
Source: J Med Internet Res. 2026 Jun 11;28:e83302. doi: 10.2196/83302 (PMC13256482; doi:10.2196/83302)
Supplement: Multimedia Appendix 5 [file jmir-v28-e83302-s005.docx]

**Multimedia Appendix 5**

**Preliminary and Finalized Versions of the mHealth Intervention for Reducing Sedentary Behavior in Older Adults**

| **Intervention Element** | **Intervention (Preliminary Version)** | **Intervention (Final Version)** |
| --- | --- | --- |
| Intervener | Geriatric, rehabilitation department medical staff, sports science experts, fitness coaches, etc. | Geriatric and rehabilitation department medical staff, sports science experts, trained researchers, etc. |
| Intervention Goal | Reduce older adults' total sedentary behavior time (≤8 hours) and screen time (≤3 hours). Note: Referencing the Canadian 24-Hour Movement Guidelines. | Reduce older adults' total sedentary behavior time (≤8 hours) and screen time (≤3 hours). |
| Intervention Duration | 12 weeks. | 12 weeks (Daily follow-up weeks 1-4 → Twice-weekly follow-up weeks 5-8 → Once-weekly consolidation/maintenance weeks 9-12). |
| Intervention Format | WeChat Mini Program and Metawear wearable device. | APP and wearable devices |
| Intervention Effect Evaluation | Objective sedentary behavior duration, frequency, count; Screen time; Activity time; Login time and frequency, etc. | Primary Outcomes: Objective sedentary behavior duration, frequency, count; Subjective sedentary behavior (Screen time); Secondary Outcomes: Activity time; Motivation for sedentary behavior and physical activity; Health literacy, Socioeconomic status, Social support; Waist circumference; Weight; Blood pressure; Fasting blood glucose; Triglycerides; HDL cholesterol; Total cholesterol; HbA1c; Quality of life; Login time and frequency; Dropout reasons; Satisfaction. |
| Intervention Content | 1. Provide information via the platform on the definition of sedentary behavior, health risks, and benefits of interrupting sedentary behavior. | 1. Provide information via the platform on an overview of sedentary behavior, health risks, and benefits of interrupting sedentary behavior. |
|  | 2. Provide instructions via the platform on how to interrupt sedentary behavior (e.g. standing, walking). |  |
|  | 3. Provide a "Sedentary Interruption Guide" via the platform, including illustrated/text tutorials for 10 simple stretching exercises (e.g."Standing Leg Stretch"), users can learn directly within the app. |  |
|  |  | 2. Reference content from sedentary guidelines of organizations like WHO via the platform. |
|  | 4. Provide information via the platform on methods for interrupting sedentary behavior. | 3. Provide information via the platform on methods for interrupting sedentary behavior. |
|  | 5. Provide information via the platform on the adverse health consequences of sedentary behavior and the benefits of interrupting it. | 4. Provide information via the platform on sedentary behavior in different scenarios and its health risks, such as watching TV in entertainment scenarios, sedentary office work in work scenarios, and the potential increase in sedentary behavior with age, explaining the adverse health consequences these behaviors may cause. |
|  | 6. Collaborate with health coaches or other professionals to assess barriers (e.g.physical limitations) and identify solutions to break sedentary behavior. | 5. Collaborate with rehabilitation physicians or other professionals to assess physical limitations (e.g.restricted joint mobility, lumbar pain, post-operative recovery), and identify solutions (e.g.push customized actions based on health data) to break sedentary behavior, adding safety prompts such as "Stop if pain occurs". |
|  | 7. Encourage individuals via the platform to accurately and repeatedly practice interrupting sedentary behavior. | 6. Help individuals accurately and repeatedly practice interrupting sedentary behavior via the platform by triggering a sedentary reminder every 30 minutes, practicing repeatedly until proficient in sedentary interruption skills. |
|  | 8. Remind users via the platform at preset times (e.g.every 30 minutes) to stand up and interrupt sedentary behavior, synchronously sending a notification "You've been sitting for 30 minutes, get up and move!". | 7. Remind users to stand up and interrupt sedentary behavior based on their preferred sedentary interruption frequency setting (15-60 minutes) via the platform, synchronously sending notifications like "You've been sitting for 30 minutes, get up and move!". |
|  | 9. Provide instructions via the platform on how to interrupt sedentary behavior and on setting up and using the application. | 8. Provide information via the platform on methods for interrupting sedentary behavior (e.g.standing, walking) and their rationale, and instructions on setting up and using the application. |
|  | 10. Provide exemplary cases via the platform of regularly interrupting sedentary behavior, to demonstrate how to accurately and correctly interrupt sedentary behavior. | 9. Provide short video exemplary cases via the platform of regularly interrupting sedentary behavior (e.g.standing stretch during TV ad breaks; standing activity during long mahjong sessions; getting up for water during long sedentary work), demonstrating how to accurately and correctly interrupt sedentary behavior. |
|  | 11. Provide feedback via the platform on areas for improvement in the individual's interruption of sedentary behavior. | 10. The platform monitors user sedentary behavior in real-time (including single-session sedentary duration and daily cumulative sedentary time) via smart wearable devices, automatically generates visual reports, and pushes personalized improvement suggestions. E.g.: Monitoring shows your longest continuous sedentary period was 14:00-16:00. Current cumulative sedentary time has reached 7 hours (approaching the 8-hour guideline limit). Suggestion: Get up and move for 5 minutes immediately; Avoid exceeding 8 hours sedentary time for the rest of today. Automatically provides feedback upon detecting user activity: Great! You have been active for 5 minutes, please keep it up! |
|  | 12. Push information via the platform on the benefits of interrupting sedentary behavior, emphasizing the necessity for older adults to receive training. | 11. Push information via the platform on the benefits of interrupting sedentary behavior, emphasizing the necessity for users to receive training. |
|  | 13. Remind individuals via the platform to arrange time reasonably to participate in training to reduce sedentary behavior, e.g.suggest individuals reduce entertainment time to decrease sedentary behavior. | 12. Remind individuals via the platform (e.g.during post-meal rest time) to participate in training (e.g."You can learn standing stretches now"). |
|  | 14. Invite a team including doctors, rehabilitation therapists, fitness coaches, etc., to provide online Q&A, offering professional advice and guidance to individuals on reducing sedentary behavior. | 13. Invite a team including geriatric doctors, rehabilitation therapists, etc., to provide online Q&A, offering professional advice and guidance to individuals on reducing sedentary behavior. |
|  | 15. Suggest via the platform that individuals invite family, friends, or colleagues to interrupt sedentary behavior together, and encourage each other through the platform's chat function. | 14. Users invite family, friends, or colleagues to interrupt sedentary behavior together via the platform, and encourage each other through the platform's chat function; family members receive reminders and send encouraging messages. |
|  | 16. Suggest via the platform that individuals make friends with people who have low sedentary behavior. | 15. The platform intelligently matches users with "partners with low sedentary behavior" based on user behavior data, and supports group challenge competitions (e.g.daily cumulative sedentary/standing time PK). |
|  |  | 16. Popularize available social support resources (e.g.senior activity centers, interest groups) via the platform, explaining the positive role of participating in these social activities in reducing sedentary behavior, guiding them to proactively seek and utilize related resources. |
|  |  | 17. Disseminate information about available environmental resources (e.g.community fitness facilities) through the platform, explaining their positive role in reducing sedentary behavior, and guide individuals to proactively access and utilize these resources. |
|  | 17. Automatically record the type, time, and frequency of sedentary behavior via the platform's built-in accelerometer, generating an hourly sedentary heatmap, allowing users to view details like "Most sedentary between 2-4 PM". | 18. Automatically record the type, time, and frequency of sedentary behavior via wearable devices. |
|  | 18. Provide videos via the platform featuring healthcare professionals emphasizing the benefits of interrupting sedentary behavior. | 19. Provide videos via the platform featuring healthcare professionals emphasizing the benefits of interrupting sedentary behavior. |
|  | 19. Display the user's ranking of sedentary behavior duration within their friend circle via the platform's "Leaderboard" function, motivating users to reduce sedentary behavior to improve their ranking. | 20. Display the user's progress ranking for sedentary behavior duration within their friend circle via the platform's "Progress Leaderboard" function, motivating users to reduce sedentary behavior; non-completers receive a "Try again tomorrow" encouragement card. |
|  | 20. After achieving sedentary behavior reduction goals, users can unlock achievement badges (e.g."Sedentary Slayer") and share them on social media to receive likes and encouragement from friends. | 21. The platform intelligently pushes layered incentives based on user goal achievement (completers unlock badges like "Sedentary Slayer" or "Star of Vitality" + voice congratulatory message; non-completers receive a "Try again tomorrow" encouragement card) and share them on social media to receive likes and encouragement from friends. |
|  | 21. On the platform, introduce the benefits of interrupting sedentary behavior through videos or health consultations. |  |
|  | 22. Reference content from sedentary guidelines of organizations like WHO (e.g."At least 1 minute of activity per hour") in the platform's sedentary classroom module to enhance user trust. |  |
|  | 23. Require individuals via the platform to use words such as "strongly," "commit," or "high priority," "I will" to confirm or reaffirm their commitment to starting, continuing, or restarting attempts to improve sedentary behavior. | 22. Require individuals via the platform to use words such as "willing," "can," "commit," or "definitely can," "high priority" to confirm or reaffirm their commitment to starting, continuing, or restarting attempts to improve sedentary behavior. |
|  | 24. Sign a sedentary commitment contract with individuals via the platform to ensure regular interruption of sedentary behavior. | 23. Sign a sedentary behavior commitment contract with individuals via the platform to ensure regular interruption of sedentary behavior. |
|  | 25. Set sedentary behavior reduction goals (e.g."Daily sedentary time ≤ 8 hours") via the platform. | 24. Set sedentary behavior reduction goals (e.g."Daily sedentary time ≤ 8 hours") via the platform. |
|  | 26. Support creation of "Sedentary Interruption Plans" via the platform, e.g."Perform a 5-minute standing stretch at 10:00, 14:00, 16:00 each day", with reminders via the platform. | 25. Support creation of "Sedentary Behavior Interruption Plans" via the platform, e.g."Perform a 5-minute standing stretch at 10:00, 14:00, 16:00 each day", with reminders via the platform. |
|  | 27. Generate weekly sedentary data reports, comparing actual sedentary time with goals. Users can view historical records via the platform's timeline function to assess goal achievement progress. | 26. Generate weekly sedentary behavior data reports, comparing actual sedentary behavior time with goals. Users can view historical records via the platform's timeline function to assess goal achievement progress. |
|  | 28. Provide specific information via the platform on the health risks of sedentary behavior (e.g."Each additional hour of sedentary time increases diabetes risk by 9%"). |  |
|  | 29. Introduce via the platform how reducing sedentary behavior can alleviate individuals' negative emotions. | 27. Introduce via the platform how reducing sedentary behavior can alleviate individuals' negative emotions. |
